# Comparative analysis of selected exhaled breath biomarkers obtained with two different temperature-controlled devices

**DOI:** 10.1186/1471-2466-9-48

**Published:** 2009-11-30

**Authors:** Frank Hoffmeyer, Monika Raulf-Heimsoth, Volker Harth, Jürgen Bünger, Thomas Brüning

**Affiliations:** 1 BGFA, Research Institute of Occupational Medicine German Social Accident Insurance, Ruhr-University Bochum, Buerkle-de-la-Camp Platz 1, 44789 Bochum, Germany

## Abstract

**Background:**

The collection of exhaled breath condensate (EBC) is a suitable and non-invasive method for evaluation of airway inflammation. Several studies indicate that the composition of the condensate and the recovery of biomarkers are affected by physical characteristics of the condensing device and collecting circumstances. Additionally, there is an apparent influence of the condensing temperature, and often the level of detection of the assay is a limiting factor. The ECoScreen2 device is a new, partly single-use disposable system designed for studying different lung compartments.

**Methods:**

EBC samples were collected from 16 healthy non-smokers by using the two commercially available devices ECoScreen2 and ECoScreen at a controlled temperature of -20°C. EBC volume, pH, NOx, LTB_4_, PGE_2_, 8-isoprostane and cys-LTs were determined.

**Results:**

EBC collected with ECoScreen2 was less acidic compared to ECoScreen. ECoScreen2 was superior concerning condensate volume and detection of biomarkers, as more samples were above the detection limit (LTB_4 _and PGE_2_) or showed higher concentrations (8-isoprostane). However, NOx was detected only in EBC sampled by ECoScreen.

**Conclusion:**

ECoScreen2 in combination with mediator specific enzyme immunoassays may be suitable for measurement of different biomarkers. Using this equipment, patterns of markers can be assessed that are likely to reflect the complex pathophysiological processes in inflammatory respiratory disease.

## Background

Exhaled breath condensate (EBC) is the liquid phase of the respiratory air sampled by cooling and is mainly formed by water vapour, but volatile substances in gas phase as well as non-volatile compounds, such as proteins carried in droplets can dissolve in condensed water during the sampling [1]. Collection of EBC is a non-invasive tool for assessing pathophysiologic processes in airway diseases [2]. Since EBC contains no cellular components the evaluation and quantification of airway or lung pathology is based on detection of biomarkers [3]. Most of them were already referred to in induced sputum or BAL and both airway and alveolar compartments contribute to the formation of EBC [4].

However, there are still many methodological limitations, and the interpretation of findings is hampered by the fact that the most widely used devices differ significantly in collection efficiency of markers of interest [5]. There might be an optimal sampling condition for every mediator. However, it is obvious that one collection technique will not be optimal for all compounds of interest using EBC as matrix. Therefore, when studying different biomarkers in one EBC sample, the methodical setting often is based on a compromise and should be appropriately evaluated [6]. This is also true for the analytical assays concerning sensitivity and specificity versus availability, cost, and technician time required [7].

Nowadays, different devices are commercially available, including (in alphabetical order) Anacon (Biostec, Valencia, Spain), ECoScreen (Cardinal Health, Hoechberg, Germany), RTube (Respiratory Research, Charlottesville, VA, USA), and TURBO-DECCS (ItalChill Pharma and Incofar Srl, Modena, Italy) [8].

Recent studies highlighted that physical characteristics of the condensing device affect the biomarker recovery in EBC. Different adhesion capacities may partly account for the disparity in the results obtained with different devises. EBC pH-values obtained with the RTube collection device were more acidic than those provided by ECoScreen [9]. In healthy volunteers, LTB_4 _could not be detected in any sample using immunoassays while cysteinyl-LT (cys-LT) was present in samples gained by ECoScreen, but not when RTube or Anacon were used as condensers [10]. In another study Cys-LT could be quantified by using EIA kits in EBC samples of RTube and ECoScreen [11]. Influences of the condensation equipment were also demonstrated for collection of 8-isoprostane and albumin [12] or oxides of nitrogen (NOx), total protein, mucin and pH [13]. Recently, reproducibility of hydrogen peroxide, 8-isoprostane and cytokines in EBC from healthy adult volunteers was demonstrated to be equally variable for different condensing devices [14]. In an excellent review, reference values of most studied biomarkers were presented referring to collecting device and analytical procedures as well as data on assay reproducibility, repeatability, variability and biomarker stability in EBC samples [15].

The composition of the condensate depends amongst sampling equipments mainly on cooling temperatures. The impact of the condensing temperature on pH was demonstrated using RTube at a starting temperature of -20 or -70°C [10]. The cooling conditions differ between the widely used devices. A warm-up during condensation is observed using RTube or the Anacon device, while in the TURBO-DECCS and ECoScreen device the cooling temperature is stable [10,16].

ECoScreen is commercially available, widely used and prevents salivatory contamination of EBC. However, this device may have limitations as exhaled breath condensates on a teflon coated surface that is repeatedly used. Recently, it was reported that NOx measurements might be confounded by the device and represent (partly) a contamination with NOx originated from the device itself [13].

ECoScreen2 (FILT, Berlin, Germany) was designed with the objective to collect fractionated samples of EBC. For this purpose, the exhaled volume can be collected into two separate chambers in a breath-controlled way. Different valves separate inspiration from expiration and direct the exhaled volume according to a threshold volume into the two chambers and a dead space volume may also be discarded. According to its design, saliva contamination is highly unlikely and was excluded in extensive testing by control of the amylase activity and the viscosity.

The aim of the current study was to compare the temperature-controlled ECoScreen2 with ECoScreen. The biomarkers studied reflect their widespread application in assessing the pathophysiology of airway diseases. We evaluated inflammatory mediators as leukotriene B_4 _(LTB_4_), cysteinyl-LT (cys-LT; LTC_4_/LTD_4_/LTE_4_) and prostaglandin E_2 _(PGE_2_), the lipid peroxidation marker 8-isoprostane (8-iso-PGF_2α_) and the NO metabolites nitrate/nitrite (NO_3_^-^/NO_2_^-^). EBC pH is a result of the relative amount of different volatile and non-volatile components such as CO_2_, ammonia, inorganic ions, organic acids, and peptides [1]. It was demonstrated to be lowered in various inflammatory lung diseases and correlated with pro-inflammatory cytokines in the airways. There is general consensus regarding the utility of EBC pH as a simple integrative marker of airway inflammation [17]. To our knowledge, no comparative data have been published to date on EBC analysis based on these devices and biomarkers.

## Methods

### Subjects

Sixteen non-smoking healthy volunteers without lung diseases and occupational exposures to irritative airborne agents were recruited for the assessment of methodological factors on sampling of EBC. A self administered questionnaire concerning the previous and current medical history was answered by each subject. Subjects with significant renal, hepatic, cardiovascular disease, or cancer were excluded. All participants had no history of chronic respiratory disorders and reported no symptoms of an upper respiratory infection within the previous six weeks. There were no foci of inflammation in the oral cavity. The demographic and clinical characteristics of the study population are shown in Table 1. All volunteers underwent assessment of lung function using a MasterLab pro (Software version 4.67a, Cardinal Health, Hoechberg, Germany) within 1 day of the measurement of EBC. According to the American Thoracic Society (ATS) criteria, forced vital capacity (FVC) and forced expiratory volume in 1 sec (FEV_1_) were obtained from three acceptable lung function tests [18]. Normal lung function was defined as FEV_1 _≥ 80% predicted and an FEV_1_/FVC ratio ≥ 70%. The study was approved by the Medical Ethics Committee of the Ruhr University of Bochum and all participants gave written informed consent.

**Table 1:** Characteristics of the study subjects

	Category	N	Median	Range
Gender	Male	9		
	Female	7		
Age (years)			39	26 - 62

Smoking status	Current/Ex-smokers	0		
	Non-smokers	16		

FVC % predicted		16	108.9	93.1 - 147.2
FEV_1_% predicted		16	102.5	88.1 - 139.8
FEV_1_/FVC [%]		16	81.7	70.1 - 93.9

FVC, forced vital capacity; FEV_1_, forced expiratory volume in 1 sec.

### Collection of EBC and study design

EBC was collected in each subject between 8 and 12 a.m. No food or drinks were allowed 2 h prior to EBC collection. Before sampling the subjects were asked to rinse their mouths with water, than EBC was collected during tidal breathing while sitting comfortably. The subjects used a nose-clip, breathed through a mouthpiece and a two-way nonrebreathing valve that also prevented saliva contamination in addition to an integrated saliva trap. They were instructed to swallow excess of saliva after coming off the mouthpiece. We compared the commercially available devices ECoScreen (Cardinal Health, Hoechberg, Germany) and ECoScreen2 (FILT, Berlin, Germany) in a randomised sequential order. Those in the first group used ECoScreen in the first sampling period and ECoScreen2 in the second, while in the second group the order was reversed. EBC collections were performed at the same day with a 30 min interval between both sample collections.

ECoScreen condensated the exhaled breath on a teflon coated surface and the sample was collected in a polypropylene cap intended for single use. The teflon coated tube was washed with high-pure water and wiped clean before each test. Due to its repeated use, the effect of long term mechanical wear has to be considered and therefore a brand-new ECoScreen device was used. In contrast the ECoScreen2 device directly condensated and collected the EBC in disposable bags on a special polyethylene surface. The exact composition and primary source of supply of the polyethylene plastic film are company secret. For comparison reasons, breath parameters were defined and controlled in a way that the complete volume was sampled in only one ECoScreen2 chamber. The ECoScreen2 feature of exclusion of a volume representing the dead-space was also not used.

Minute ventilation was measured by an inserted pneumotachograph (EcoVent, Cardinal Health, Hoechberg, Germany) when using ECoScreen or by an integrated pneumotachograph in case of ECoScreen2. For each time point, EBC was collected for exactly 10 min and during the collection period a temperature of -20°C was maintained by both condenser devices.

In order to evaluate repeatability, in a preliminary study eight subjects used either ECoScreen or ECoScreen2 in a repeated way under the same conditions (pausing 30 min between the collections of EBC, similar breathing patterns) and the mean within-subject differences of EBC volume, pH and mediator concentrations in the samples were determined.

### Sample processing

After EBC collection, samples were immediately divided in aliquots and stored at -70°C. Stability of the biomarkers in the frozen samples was demonstrated before [6]. Within 2 months at -70°C the aliquoted samples were thawed and analysed for biomarkers. All EBC samples were analysed in a blinded way for the ECoScreen and ECoScreen2 device. The samples underwent only one single freeze-thaw cycle, were analysed in duplicate or triplicate and run together to exclude interassay variation.

#### Determination of pH

The pH was measured using a pH-meter with a glass-electrode (Mettler Toledo, Giessen, Germany). The range was 0.00 to 14.00 and the accuracy about 0.01 +/- 0.02. The pH was measured immediately and after gas standardisation (deaeration) that was performed with argon at 2 bar for 10 min.

#### Determination of NOx

The concentration of nitrogen oxides (NO_2_/NO_3_; NOx) in EBC was determined by a spectrophotometric assay based on the Griess reaction (Cayman Chemical Company, Ann Arbor, MI, USA), as previously described [19]. The assay was performed in triplicates of 80 μL EBC together with 10 μL enzyme cofactors and 10 μL nitrate reductase reacted with 25 μL Griess reagent. The total NO was measured at 540 nm with a microplate reader (Spectramax Molecular Devices, Sunnyvale, USA) and calculated by using a standard curve with the Softmax Pro 4.7.1, software. The range of quantification was 5 μM - 35 μM. Samples with higher concentrations were diluted.

#### Determination of LTB_4_, PGE_2_, 8-iso PGF_2α _and cys-LT

The concentrations of all four biomarkers were measured by specific competitive immunoassays (Assay Designs, Ann Arbor, USA). The kits use a polyclonal (in the case of LTB_4 _and PGE_2_) or a monoclonal (for cys-LT and 8-iso-PGF_2α_) antibody to bind the relevant marker or an alkaline phosphatase molecule, which has the biomarker covalently attached to it. After incubation and washing, the generated yellow colour is read with a microplate reader (Spectramax Molecular Devices, Sunnyvale, USA) at 405 nm. The intensity of the yellow colour is inversely proportional to the concentrations of the biomarker in either standards or samples. The optical density is used to calculate the concentration of the biomarker by using the Softmax Pro 4.7.1 software utilizing a 4-parameter logistic curve fitting program. In each assay, the lowest standard was set as the limit of quantification (LOQ) of the assay. The LOQ of LTB_4 _was 11.7 pg/mL and the maximum cross-reactivity was 5.5% for 6-trans-12-epi-LTB_4_. The LOQ of PGE_2 _was 39.1 pg/mL and the maximum cross-reactivity was 70% for PGE_1_. The LOQ of 8-iso PGF_2α _was 6.1 pg/mL and the maximum cross-reactivity was 4.6% for PGF_1α_. The LOQ of cys-LT was 78.1 pg/mL and the monoclonal antibody recognized LTD_4 _with 115.1%, LTC_4 _with 100% and LTE_4 _with 62.7%. Maximum cross-reactivity was 1.6% for LTB_4_. All values were measured in duplicate. The intra-assay variability's of the biomarkers by these specific competitive immunoassays were < 15%.

### Statistical analysis

Value distribution was assessed using the D'Agostino & Pearson omnibus normality test. Repeated measurements were analyzed as recommended by Bland and Altman [20]. In addition, repeatability was estimated as correlation and as coefficient of variation (CV = standard deviation/mean as percent), which was calculated as mean of the individual CVs of the repeated measurements.

Comparisons of paired data were performed with paired t-test or Wilcoxon test, where appropriate. Values below the LOQ were set 2/3 of LOQ. The data were analysed by using GraphPad Prism version 5.01 for Windows (GraphPad Software, San Diego, California, USA, http://www.graphpad.com). A two-sided significance level of 0.05 was chosen for all tests. Data are expressed as median with interquartile range. Differences were calculated as recommended by Bland and Altman. Correlations between parameters measured with the two devices were calculated with Pearson's test or Spearman rank test, where appropriate.

There were no evidences of an influence of the order of the devices concerning EBC volume, EBC pH and the biomarker investigated according to the statistical approach of Hills and Armitage [21].

## Results

### Repeatability

The repeated measurements of volume, pH and the mediators in EBC obtained with the same device within 30 minutes showed a different degree of repeatability. The mean differences of the two sampling periods (bias), coefficient of variation (CV) and correlations (r^2^, p and the intraclass correlation coefficient, ICC) are depicted (Tab 2). The mean concentrations of NOx were different in EBC sampled by ECoScreen in repeated periods and showed poorly reproducibility. The intraclass correlation coefficient for NOx was not calculated because NOx levels were under the LOQ in half of the subjects. Regarding LTB_4_, we noticed that in EBC obtained with ECoScreen, all samples turned out to be detectable but under the LOQ. The mean concentration was not significantly different in the repeated sampling (p = 0.92), however, there was no correlation of the two periods (p = 0.82). In contrast, concentrations of LTB_4 _in EBC samples collected with ECoScreen2 were in the linear part of the calibration curve of the competitive immunoassay. In all the samples tested for Cys-LT, no activity was detected.

**Table 2:** Results of statistical analysis on repeatability

EcoScreen						
	**Volume**	**pH**	**LTB_4_**	**NOx**	**8-iso PGF_2α_**	**PGE_2_**

bias %	3.5	0.2	^+)^	48.9	2.1	^+)^
CV %	7.7	0.9		166.3	21.4	

r^2^	0.479	0.889		n.c.	0.816	
p	0.027	< 0.0001		n.c.	0.002	
ICC	0.692	0.943		n.c.	0.903	

**ECoScreen2**						

	**Volume**	**pH**	**LTB_4_**	**NOx**	**8-iso PGF_2α_**	**PGE_2_**

bias %	0.5	0.7	9.0	^+)^	3.2	11.9
CV %	8.0	1.0	18.6		11.5	34.4

r^2^	0.618	0.915	0.656		0.626	0.704
p	0.021	0.0002	0.015		0.017	0.018
ICC	0.786	0.957	0.810		0.791	0.839

Repeated measurements of volume, pH and the mediators in EBC obtained with either the ECoScreen or ECoScreen2 device within 30 minutes. Values of the mean differences of sampling period 1 and 2 (bias), CVs and correlations. CV, coefficient of variation; ICC, intraclass correlation coefficient; n.c., not calculated. ^+)^mediator concentration < LOQ.

### EBC volume

**Figure 1:**
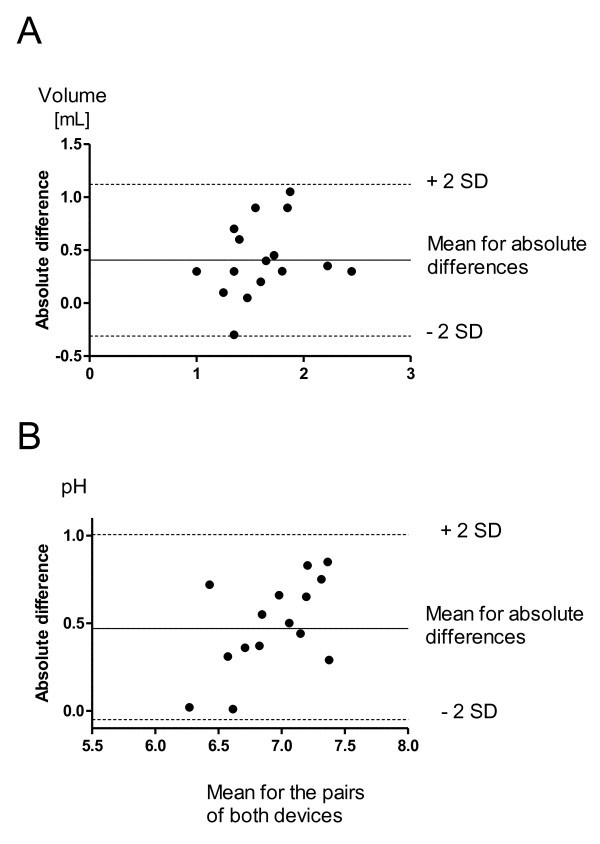
**Comparison of sample volume (A) and EBC-pH (B) obtained with the two devices**. Difference between the values (ECoScreen2 -- ECoScreen) plotted against the mean sample volume according to Bland and Altman [20]. The continuous line represents the mean difference and the dashed lines represent the ± SD for the differences.

After 10 min of tidal breathing, a significantly higher EBC volume was collected with ECoScreen2 (median 1825 μL; interquartile range 1500 - 2225 μL) than with ECoScreen (1425 (1125-1575) μL; p < 0.01). The measurements obtained with the two devices revealed a mean within-subject difference of 412 μL (SD 350 μL). All differences between the sample volumes lay within ± 2 SD (Fig 1A). Voluntary ventilations assessed by an integrated pneumotachograph (ECoScreen2) or by Eco-Vent (ECoScreen) were not significantly different (p > 0.05, data not shown).

Using ECoScreen exhaled breath condensates directly in a polypropylene cap where it is sampled. Thereby EBC presents the total sample amount. In case of ECoScreen2, the condensate has to be recovered and transferred into a cap before further sample processing is possible. There is a loss of condensate during the step of EBC harvesting (data not shown).

### EBC pH

Breath condensate collected with ECoScreen2 was less acidic than the pH of samples collected with ECoScreen when pH of EBC was measured immediately (7.24 (6.79 - 7.52) vs. 6.65 (6.42 - 6.87); p < 0.0001). We assessed a mean difference in the pH levels of 0.47 (SD 0.27) (Fig 1B). The pH values obtained after argon deaeration and storage were significantly higher than pH values immediately measured for both ECoScreen2 (7.59 (7.28 - 7.73) vs. 7.24 (6.79 - 7.52); p < 0.0001) and ECoScreen (7.05 (6.72 - 7.28) vs. 6.65 (6.42 - 6.87); p < 0.0001). The mean pH value was also significantly elevated after argon treatment (0.49, SD 0.25).

**Figure 2:**
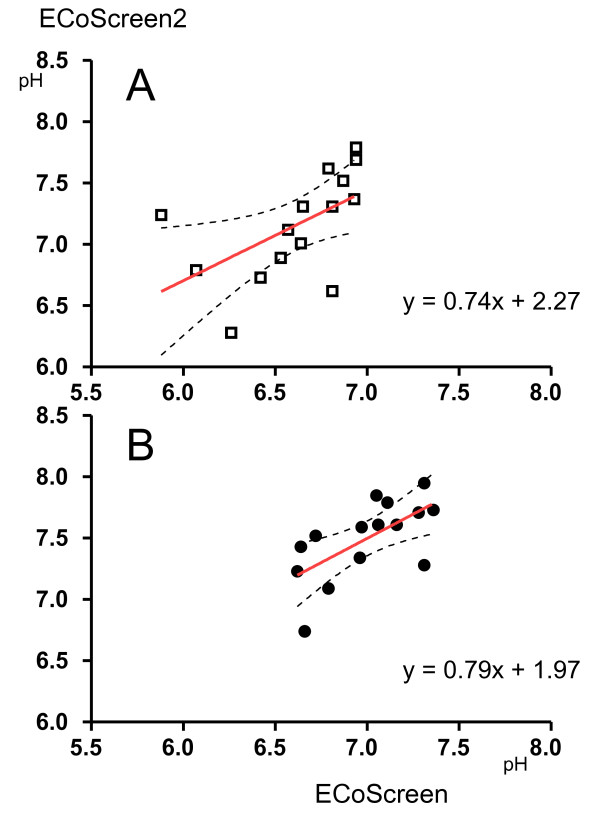
**Correlations between pH values obtained with ECoScreen (x--axis) and ECoScreen2 (y-axis)**. A: Immediate determination; B: determination after argon treatment and -70°C storage.

There was a correlation between pH values immediately determined after collection with both devices (r^2 ^= 0.56; p < 0.05) that was also apparent after argon treatment (r^2 ^= 0.65; p < 0.01). In linear regression models the correlation between the devices (ECoScreen to ECoScreen2) was almost identical for the different methodological approaches of pH measurements, e.g. immediately determined (y = 0.74x + 2.27) or after argon treatment and storage (y = 0.79x + 1.97) (Fig 2).

### Biomarkers

All patients were able to produce EBC, however sometimes the sample amounts were not sufficient to measure all biomarkers. Cys-LT could not be detected in any sample collected either with ECoScreen or ECoScreen2.

The measurements of NOx, LTB_4 _and PGE_2 _were significantly influenced by the collection device. Concentrations of NOx in EBC collected with ECoScreen were 4.3 (3.3 - 7.1) μM, but all ECoScreen2 samples were below the LOQ of the assay. On the other hand, even though in all samples LTB_4 _was demonstrated, only LTB_4 _samples generated by ECoScreen2 had substantial concentrations (22.8 (8.9 - 36.7) pg/mL). In all ECoScreen samples, LTB_4 _concentrations were detectable (about 5 pg/mL) but below the LOQ.

PGE_2 _was only detectable and above the LOQ (202.7 (55.6 - 356.1) pg/mL) when EBC was collected with ECoScreen2. 8-iso PGF_2α _could be detected and quantified in every sample. Breath condensate collected with ECoScreen2 yielded significantly higher concentrations of 8-iso PGF_2α _than ECoScreen (464.4 (223.0 - 661.1) pg/mL vs. 81.9 (70.2 - 241.5) pg/mL; p < 0.01). The levels obtained with the two devices revealed a mean within-subject difference of 275 pg/mL (SD 246 pg/mL). There was no correlation between values collected with both devices (r = 0.27).

The relative amount of biomarkers above the LOQ and their values obtained by the different collection devices are shown in (Table 2).

## Discussion

EBC is an attractive non-invasive method for assessing airway diseases but its clinical use is still hampered by methodical limitations [5]. There is accumulating evidence that the physical surface properties of the collecting devices influence the marker measurement [9,12-14,22]. Moreover, for some markers the applied temperature was identified as a major confounder [10,11].

The objective of this study was to evaluate the new commercially available temperature-controlled ECoScreen2 in comparison to the commonly used collecting system ECoScreen. The ECoScreen2 device was designed for studying different lung compartments. It enables breath-controlled collection into two separate chambers reflecting different depths of the bronchial system (the airways or the alveoli).

EBC samples collected by each device were compared on the same subject in a cross-over trial during two sampling periods. Subjects were allocated randomly to two different groups and EBC collection was performed for each device at -20°C according to general methodological recommendations on the collection and analysis of EBC [5]. No influence of the order on the measurements could be identified. In addition, effects from both time and carry over effects from the previously sampling period were not detectable when the same device was repeatedly used. This underlines the concept of EBC as a non-invasive method and the usefulness of this method for repeated sampling. In contrast to invasive methods like BAL or sputum induction there is no reasonable mechanism by which collection of EBC might significantly alters the airway lining fluid. Our randomization design was in line with previous reports addressing comparison of different collecting devices [9,11-14] or condensation temperatures in a sequential manner [10,16]. Usually, a short time interval of 5 to 15 minutes after every collection was chosen [9,16,23] but there were also trials without any break [10]. We choose a time period of 30 min for the subjects to relax and to concentrate on a regular calm tidal breathing during the second period. Under this condition the subjects managed to breath with a quite similar breathing pattern during repeated sampling of EBC. Confounding was further reduced by applying both methods ante meridiem.

In preliminary studies the repeatability of both devices was also assessed under similar conditions. Talking about variability, it is to stress that in the chosen setting the total variability has to be taken into account. That includes intra-subject variability, technical factors of collection and analytical impact. Intra-subject variability was minimized by performing both sampling periods sequentially. Collecting of EBC was standardised according to a fixed time, temperature and breathing pattern. Under these conditions, we could demonstrate acceptable coefficients of variation concerning the volume and EBC-pH. Reproducibility of volume and pH was comparable for both devices and in accordance with recent findings [14]. Hence, we do believe that one major reason for the higher variability in biomarker levels was due to the analytical assays that add most variability to the overall variability. Referring to LTB_4_, we could demonstrate that concentrations of mediators within the LOQ favour reproducibility. Therefore choosing a device that enables a detection of concentrations above the LOQ may lead to an improvement of biomarker reproducibility. Enhanced mediator concentrations avoid higher standard deviation in immunoassays [7]. Reproducibility expressed as coefficient of variation was in the line with recently published data [14,23].

We found that collecting EBC at -20°C resulted in a higher sample volume with ECoScreen2 than with ECoScreen. Since the duration of collection for both devices was exactly 10 min and breathing patterns (measured by pneumotachograph) were similar, the observed differences should reflect the different condensing materials. An influence of the surface material on the efficiency of condensation was reported earlier [13]. Moreover, it is obvious that an enlarged condensation surface favours sampling of an increased EBC volume [14]. Therefore, the higher sampling volume in case of ECoScreen2 might be at least attributed to its higher condensing surface. The volume of the primary condensate should be considered even higher because the condensate is dispersed to the high surface of the bags and has to be harvested. Consequently, a reversal is obvious between the primary condensate amount and the sample that could be used for further analysis. In case of ECoScreen, the total amount of the condensate is sampled in a cap enabling immediate processing without further volume loss.

Published data reveal that EBC pH is correlated with other marker for airway inflammation. Low EBC pH in several respiratory diseases pointing towards the important pathophysiologic role of airway acidic stress [17]. The determination of pH with a glass-electrode is an established accurate method. However, there are different recommendations concerning the general conditions of EBC pH measurements (immediate measurement vs. after deaeration with a CO_2_-free gas, mostly argon) as it was shown that EBC pH is influenced by the CO_2 _level. EBC pH measurements after deaeration with argon were found not to be affected by collection temperatures [24]. When using a different method with determination of pH at a normalized standard partial pressure, a change of the condensing temperature in the same device was shown to influence the EBC pH, and samples collected at lower temperature were more acidic [10]. This technique for pH measurement was recently introduced promising a further improvement of the reproducibility [25]. In the present study, pH values were assessed before and after deaeration. Our general focus was the application of EBC analysis in field studies and therefore we chose not to determine pH at a normalized standard partial pressure as this procedure needs more equipment and is time-consuming. The methods used in this study for pH measurement have been validated and were previously shown to be robust and reliable [9,13,24]. Normative data were characterized in 404 healthy subjects [26]. Our findings of EBC pH values measured immediately after collection or with deaeration corroborate our previous results and are in accordance to the published data referring to ECoScreen [6,15].

There are only limited data from comparative studies concerning the methodical impact. In a heterogeneous study population, higher pH values were reported for ECoScreen than RTube regarding non-deaerated EBC samples. With deaeration the difference was smaller and near to insignificance [9]. In another study there was no difference between pH assessed after deaeration in EBC samples from healthy individuals collected with the ECoScreen or RTube device [11]. Different pH values with a low correlation were reported between non-deaerated samples collected by ECoScreen and RTube in healthy subjects [23]. In contrast, in our comparative design we found a good correlation between pH values in non-deaerated and in deaerated EBC obtained by ECoScreen or ECoScreen2. The pH of EBC depends on a mixture of non-volatile and volatile components, CO_2 _being the major one. In our study the correlation between pH obtained with the different devices could be described by an equation that was similar for samples before and after deaeration. This result implicates that the differences of EBC pH are determined by other acids (or bases).

Stability of NO was demonstrated to be influenced by collecting temperature, which may contribute to the described variation of NOx levels in EBC [27]. Moreover, elegant control experiments unravel that the device itself can introduce errors in the measurement of markers and the NOx sampled by ECoScreen partly represents a contamination with NOx originating from the device itself [13]. In the EcoScreen, the condensation occurs on the surface of a re-useable module with modified inner Teflon coating and the resulting EBC is collected in a disposable polypropylene cap. Preliminary investigations demonstrated an effect of the age of the condensing module on NOx levels. A significant difference could be revealed when NOx was analysed in samples from the same volunteers obtained either with a 20 month-old frequently used device or a new one, (samples > LOQ 1/16 vs. 8/16, data not shown). For the current study we use a new ECoScreen condenser. There was a high variability in the repearted measurements and no correlation between values obtained in the first and second period. This further supports the idea of a contamination with NOx in case of ECoScreen.

When comparing studies using the same device, the observed variability of biomarker levels may also be attributed to an analytical impact, especially of the currently used immunoassays. At low concentrations, assays often become nonlinear. Since results are based on interpolation, the variability of the assay and thereby the coefficient of variation (CV) increases. Therefore, it is not surprising that mean CV's are high when biomarker levels are close to the detection level of the assay. Our comparison is based on the LOQ of the assay rather than on the lower limit of detection [7,28]. We chose different biomarkers for analysis that reflect different aspects of inflammation including oxidative stress. Parallel identification of different biomarkers is a prerequisite for the evaluation of special biomarker profiles, which might be helpful in differential diagnosis or assessment of disease severity [29].

Immunoreactivity for LTB_4 _was reported in the majority of healthy subjects when EBC was sampled with ECoScreen. Our results are in line with previous investigations reporting LTB_4 _levels in the range of 3.8. to 7.7 pg/mL and close to the limit of detection [22,30]. Despite the use of a comparable methodology some studies demonstrated rather high LTB_4 _levels in EBC of healthy subjects [31,32]. In contrast, no LTB_4 _could be detected in any sample collected with ECoScreen in a recent study designed to evaluate the influence of condensing equipment on leukotriene concentrations [10]. With the specific enzyme immunoassay applied in our study the concentrations of LTB_4 _were in most samples within the limit of quantification using ECoScreen2 and significantly higher compared to collection with ECoScreen.

Cys-LTs were not in the range of detection in any of our samples, regardless of the condenser type. Cys-LTs measurements were reported to be measurable in healthy volunteers; however their levels were often near or even below the LOD of the applied EIA when using ECoScreen for EBC sampling [22,32]. In two studies higher concentrations of Cys-LTs were detected with a commercially available EIA kit in samples from ECoScreen compared with RTube [10,11]. Our methodology was different as another Cys-LT EIA kit was used that was demonstrated in our laboratory to be effective in fluids like nasal lavage or sputum. Referring to published data, Cys-LTs concentrations in EBC were reported up to 205 pg/mL [11]. However, in all the samples tested for Cys-LT, no activity was detected, suggesting that the sensitivity of our assay (LOQ: 78.1 pg/mL) was not appropriate for the detection of Cys-LTs in EBC regardless of the type of device used. This is consistent with previous findings demonstrating Cys-LT concentrations rather in the range of 15-25 pg/mL [32,33] or 65 pg/mL [10].

Data on PGE_2 _levels in EBC are limited. Considering the anti-inflammatory effects of PGE_2 _this mediator might be valuable for evaluating endogenous balance in inflammatory diseases. In most subjects of our study, PGE_2 _levels were detectable and in the linear range enabling valid quantification when using ECoScreen2 for EBC sampling and EIA for its measurement.

8-iso PGF_2α _belongs to the group of F_2_-isoprostanes and reflects oxidative stress and lipid peroxidation [34]. In healthy subjects a base-line level of 8-isoprostane could be verified and an influence of the condensing surface was demonstrated [12,14]. Compared to these results and a previous study on healthy smokers using ECoScreen, which reported a detectability in about half of the subjects [35], we could detect 8-iso PGF_2α _in all samples with high immunoassay activity. We conclude that there is a substantial impact of the condensing material and ECoScreen2 was superior resulting in significantly higher levels of 8-iso PGF_2α _using the same EIA kit. There was no correlation for the 8-iso PGF_2α _concentrations between both devices.

ECoScreen2 is a temperature-controlled device and in contrast to ECoScreen it is a single-use disposable condensing and collection system. Compared with the Teflon-coated metal surface of ECoScreen the plastic collecting surface of ECoScreen2 turned out to be more appropriate for lipid-derived compounds as stated earlier [8]. This device might be useful for studying different lung compartments as it allows breath controlled collection of exhaled breath derived from the airways or the alveoli into two separate chambers were the respective EBC is sampled. In summary, ECoScreen2 was superior concerning condensate volume and detection of specific biomarkers. The number of samples showing values above the LOQ of LTB_4 _or PGE_2 _was higher than in ECoScreen. The ECoScreen2 condenser was also associated with significantly higher concentrations of 8-iso PGF_2α_.

Variations in biomarker detection in favour of ECoScreen2 could not simply be attributed to variations in the dilution as the quantity of condensate was also significantly increased. We did not quantify the dilution of the EBC samples. It was suggested that epithelial lining fluid concentrations could be calculated from EBC values by using dilutional indicators like urea, ion measurements or total protein but it is to note that they are also a source of variability [1,5]. Dilution markers might be unnecessary as ratios of biomarkers are determined. In this respect, the EBC pH can be considered as a ratio of acids and bases and pH was the most reproducible marker. Moreover, there was a strong correlation between the values determined by both devices.

## Conclusion

Each biomarker has its own chemical and physical characteristics. It was suggested that the most valid and inert condenser coating should be identified and applied for the measurement of a specific inflammatory marker. On the other hand, specific patterns of markers are likely to reflect more accurately the complex pathophysiological processes in respiratory disease, since markers of airway inflammation are differently expressed in different diseases as well as disease severities. The current results demonstrate that ECoScreen2 is a suitable collecting device that enables detection of different biomarkers that reflect different aspects of inflammation including oxidative stress. In addition, the detection of multiple biomarkers is a condition precedent to calculate ratios that could strengthen interpretation of changes according to airway diseases. Further studies are needed to confirm our findings and it would be of particular interest to report concentrations of inflammatory markers in fractionated exhaled breath condensate.

## Competing interests

The authors declare that they have no competing interests.

## Authors' contributions

FH and MRH had the idea for the study, directed the study design and the data collection, and had substantial contributions in interpretation of data and writing of the manuscript. VH directed the study design and the data collection. JB and TB had contributions in interpretation of data and/or writing of the manuscript.

All authors had the opportunity to read and amend this draft version of the manuscript.

**Table 3:** Concentrations of selected biomarkers in samples collected with ECoScreen and ECoScreen2

	ECoScreen	ECoScreen2
**NOx**		
Samples > LOQ/total samples	8/16	0/16
concentration (μM)	4.3 (3.3 - 7.1)	

**LTB_4_**		
Samples > LOQ/total samples	2/16	12/16
concentration (pg/mL)	n.c.	22.8 (8.9 - 36.7)

**PGE_2_**		
Samples > LOQ/total samples	0/11	13/15
concentration (pg/mL)		202.7 (55.6 - 356.1)

**cysLT**		
Samples > LOQ/total samples	0/8	0/9

**8-isoPGF_2α_**		
Samples > LOQ/total samples	12/12	15/15
concentration (pg/mL)	81.9 (70.2 - 241.5)	464.4 (223.0 - 661.1)
Difference	p < 0.01	

NOx, nitrogen oxides; LTB_4_, leukotriene B_4_; PGE_2_, prostaglandin E_2_; cys-LT, cysteinyl-LT; 8-iso-PGF_2α_, 8-isoprostane. n.c., not calculated.

## Pre-publication history

The pre-publication history for this paper can be accessed here:

http://www.biomedcentral.com/1471-2466/9/48/prepub
